# Radiation dose-fractionation in adult *Aedes aegypti* mosquitoes[Fn FN1]

**DOI:** 10.1051/parasite/2023005

**Published:** 2023-02-10

**Authors:** Hanano Yamada, Hamidou Maïga, Carina Kraupa, Nanwintoum Séverin Bimbilé Somda, Wadaka Mamai, Thomas Wallner, Jeremy Bouyer

**Affiliations:** Insect Pest Control Laboratory, Joint FAO/IAEA Centre of Nuclear Techniques in Food and Agriculture, International Atomic Energy Agency PO Box 100 1400 Vienna Austria

**Keywords:** Irradiation, Induced sterility, Flight ability, Competitiveness, Rhodamine B, Sterile insect technique

## Abstract

Balancing process efficiency and adult sterile male biological quality is one of the challenges in the success of the sterile insect technique (SIT) against insect pest populations. For the SIT against mosquitoes, many stress factors need to be taken into consideration when producing sterile males that require high biological quality to remain competitive once released in the field. Pressures of mass rearing, sex sorting, irradiation treatments, packing, transport and release including handling procedures for each step, add to the overall stress budget of the sterile male post-release. Optimizing the irradiation step to achieve maximum sterility while keeping off-target somatic damage to a minimum can significantly improve male mating competitiveness. It is therefore worth examining various protocols that have been found to be effective in other insect species, such as dose fractionation. A fully sterilizing dose of 70 Gy was administered to *Aedes aegypti* males as one acute dose or fractionated into either two equal doses of 35 Gy, or one low dose of 10 Gy followed by a second dose of 60 Gy. The two doses were separated by either 1- or 2-day intervals. Longevity, flight ability, and mating competitiveness tests were performed to identify beneficial effects of the various treatments. Positive effects of fractionating dose were seen in terms of male longevity and mating competitiveness. Although applying split doses generally improved male quality parameters, the benefits may not outweigh the added labor in SIT programmes for the management of mosquito vectors.

## Abstract (fr)

Équilibrer l’efficacité du processus et la qualité biologique des mâles adultes stériles est l’un des défis du succès de la technique des insectes stériles (TIS) contre les populations d’insectes nuisibles. Pour la TIS contre les moustiques, de nombreux facteurs de stress sont à prendre en compte lors de la production de mâles stériles qui nécessitent une haute qualité biologique pour rester compétitifs une fois relâchés au champ. Les pressions de l’élevage en masse, du triage par sexe, des traitements d’irradiation, de l’emballage, du transport et de la libération, y compris les procédures de manipulation pour chaque étape, s’ajoutent au budget de stress global du mâle stérile après la libération. L’optimisation de l’étape d’irradiation pour atteindre une stérilité maximale tout en minimisant les dommages somatiques hors cible peut améliorer considérablement la compétitivité de l’accouplement des mâles et il est donc important d’examiner divers protocoles qui se sont révélés efficaces chez d’autres espèces d’insectes, comme le fractionnement de dose. Une dose entièrement stérilisante de 70 Gy a été administrée aux mâles *Aedes aegypti* en une dose unique ou fractionnée en deux doses égales de 35 Gy, ou une faible dose de 10 Gy suivie d’une seconde dose de 60 Gy. Les deux doses étaient séparées par des intervalles de 1 ou 2 jours. Des tests de longévité, d’aptitude au vol et de compétitivité à l’accouplement ont été réalisés pour identifier les effets bénéfiques des différents traitements. Des effets positifs de la dose de fractionnement ont été observés en termes de longévité des mâles et de compétitivité à l’accouplement. Bien que l’application de doses fractionnées améliore généralement les paramètres de qualité des mâles, les avantages peuvent ne pas compenser le travail supplémentaire dans les programmes TIS pour la gestion des moustiques vecteurs.

## Introduction

Combatting mosquito species responsible for transmitting debilitating diseases to humans and animals has been a continuous challenge throughout history. Although undeniably, the development of insecticides and repellents was a major breakthrough and has been a powerful tool against mosquito vectors to date, many of the target species have evolved to develop insecticide resistance to most of the available chemicals [28, 30, 31, 38]. Furthermore, the extensive use of insecticides comes with detrimental adverse effects in people, animals, off-target and beneficial insects, and the environment [32]. The sterile insect technique (SIT) offers an alternative, “green”, species-specific and sustainable tool for the management of insect pests and reduces the dependence on insecticide use [11].

The Food and Agriculture Organization/International Atomic Energy Agency (FAO/IAEA) Insect Pest Control Laboratory in Seibersdorf, Austria is currently tailoring the SIT for its implementation against important human disease vectors, in particular *Aedes aegypti*, *Ae. albopictus* (major vectors of dengue, chikungunya, Zika, and numerous other arboviruses) and *Anopheles arabiensis*, an important vector of malaria. This includes the development of equipment, methods and guidelines for colonizing and mass rearing the target species, sex separation, sterilization by irradiation, handling, transport and release methods, executing field trials, and quality control (QC), of which the most notable advancements are reviewed in Vreysen et al. [36].

One of the challenges in the SIT for mosquitoes is balancing sterile male production efficiency with downstream sterile male quality. Increasing stress factors such as excessive handling, selective pressures of mass rearing, external stressors like irradiation exposure, chilling and packing are among the numerous sources of stress for the mosquitoes, and these can influence the overall male quality. A high level of biological quality in the sterile males is required for their success in the field once released. The factory-produced sterile males must outcompete their wild counterparts to mate with wild females. Only then will the target population decline with each successive generation [23, 24].

It is still unclear which stress factors are most important in reducing male quality, and what combinations of stress factors may further exacerbate this. Several factors known to cause a decline in male quality indicators have been investigated, such as the pressures of mass rearing [3], chilling and packing adults [6, 7, 42], hypoxic environments, for example, during irradiation procedures [39], irradiation exposure itself [18], and a combination of factors encountered during sterile male production [8, 34, 41]. Contrarily, some studies have shown that improving handling protocols can also improve male quality. Irradiation procedures including the preparation and handling methods, and the radiation exposure itself can decrease male quality if the males are overdosed, or if handling becomes excessive, and other stress factors such as chilling, and transportation are added [9]. On the other hand, improving irradiation protocols, such as performing the exposures in hypoxia or fractionating the total sterilizing dose into two or more smaller doses have been shown to greatly improve sterile male quality in various insect species: for example, dose fractionation improved longevity in boll weevils [21]; improved competitiveness was reported in the spotted bollworm after fractionated doses, whereas longevity and insemination capacity did not change. In the Indian meal moth, however, splitting the irradiation dose into three fractions improved longevity and mating propensity [5]. Fractionating a fully sterilizing dose in the West Indian sweet potato weevil maintained competitiveness for 12 days as opposed to just 6 days when given an acute dose [25]. Ducoff et al. [10] reported that the more the irradiation dose is fractionated, the better the survival in the confused flower beetle, and fractionating dose in the presence of nitrogen greatly improved tsetse fly longevity [35].

In this study, we investigated whether fractionating the irradiation dose needed to achieve > 99% sterility in *Ae. aegypti* (70 Gy in our setting), can improve male quality in *Aedes* mosquitoes. The total dose was split either into two equal units (35 + 35 Gy) or by “conditioning” the males with a low dose of 10 Gy, followed by the additional 60 Gy. A rest period of 1 or 2 days between exposures was also tested to see whether either would result in beneficial effects on longevity, flight ability, and mating competitiveness.

## Materials and methods

### Mosquito strains and rearing

A standard laboratory reference strain of *Ae. aegypti* [12, 14] was used for all experiments. The *Aedes* strain has been maintained following the “Guidelines for Routine Colony Maintenance of *Aedes* mosquito species” (FAO/IAEA, [12]).

### Sample preparation

Pupae were collected and sexed based on pupal size dimorphism using a glass pupal sorter [16] and sex was verified under a stereomicroscope. Males were kept for treatment and females were placed in individual drosophila tubes for emergence to ensure virginity for later mating.

Adult males that emerged within a 12 h window were collected, batched in groups of 20, and kept in 15 × 15 × 15 cm Bugdorm^®^ cages (MegaView Science Co. Ltd., Taichung 40762, Taiwan) until the following day when they were briefly knocked down in a cold room at 4 °C, transferred to, and irradiated in small 2 cL plastic cups closed with a sponge. At the time of the (first) irradiation, the adults were 24–36 h old.

### Irradiation and dosimetry

Radiation treatments were performed in a Gammacell 220 (Nordion Ltd, Kanata, ON, Canada), which had a dose-rate of 59.1 Gy/min at the time of the experiment.

The dosimetry system used to verify the dose received by the samples was based on Gafchromic HD-MD-V3 film (Ashland Advanced Materials, Bridgewater NJ, USA) following the IAEA protocol [20]. Three films of MD film were packed in small (2 × 2 cm) paper envelopes and placed directly above and below the mosquito samples. Films were read with an optical density reader (DoseReader 4, RadGen, H-1118 Budapest, Sasadi út 36, Hungary) after 24 h of development.

A total dose of 70 Gy was applied for the experiments, expecting to achieve > 99% sterility, following previous irradiation dose-response experiments with this strain and irradiator [39]. Control groups were handled in the same way but were not irradiated (group A). Irradiation doses were applied to samples as follows: either an acute dose of 70 Gy (group B), or fractionated into 2 doses of 35 + 35 Gy, with either 1 day (group C) or 2 days (group D) of rest between exposures, and 10 + 60 Gy, with either 1 day (group E) or 2 days of rest (group F) between exposures (Table 1). Two biological repetitions with three technical repeats each were performed for each treatment and control group.

**Table 1:** Treatment groups, exposure intervals, and doses used (Gy).

Group	Interval duration	Irradiation dose(s)
A	Non-irradiated	0 Gy
B	Acute dose	70 Gy
C	1 day	35 + 35 Gy
D	2 days	35 + 35 Gy
E	1 day	10 + 60 Gy
F	2 days	10 + 60 Gy

### Assessing the dose response and male quality parameters following acute dose compared to fractionated doses with either a 1- or 2-day interval between exposures

#### Assessment of induced sterility

Following irradiation, the male adults were placed in 15 × 15 × 15 cm Bugdorm^®^ cages with a supply of 10% sugar solution. Twenty virgin females were added to each cage and were allowed to mate for 3 days before they were provided with 2 bloodmeals on consecutive days (days 6 and 7 post-emergence). Oviposition cups containing water and germination papers were added to each cage on day 8 for *en masse* egg collection (on days 9 and 10 post-emergence), following routine rearing protocols [12]. Egg papers were collected, matured (slow-dried over 4 days) and stored for 10 days before hatching. The total number of hatched and un-hatched eggs were counted using a stereomicroscope. Any un-hatched eggs were either opened with a dissection needle, or if many, were bleached to determine the fertility status [13].

#### Assessment of longevity

Samples of 30 adult males were reared, prepared, irradiated and caged as described above. Dead individuals were counted and removed on weekdays until all were dead. Three repetitions were performed for each treatment group and controls.

#### Assessment of flight ability

Samples of 100 (±5) adult males were reared, prepared, irradiated and caged as described above. All samples were taken to the flight test device 1 day after the last irradiation exposure. (Note: As the flight test requires that all treatment groups and control are run at the same time, and with adults of the same age, sample groups B, C and E had 2 recovery days after the last irradiation exposure and prior to the flight test, whereas groups D and F only had 1 day of rest). The flight test was performed as described in [29]. Two biological repetitions with each two technical repeats were performed for each treatment group and control.

#### Assessment of mating competitiveness

To evaluate whether fractionating irradiation dose is beneficial in terms of resulting sterile male competitiveness, and whether 1 or 2 days of rest between exposures improves male quality, and whether 2 equal half doses (35 + 35 Gy) or a low dose followed by a high dose (10 + 60 Gy) results in more competitive males, two types of sterile males were offered to virgin females for direct competition as follows: B *vs*. C, B *vs*. E, C *vs*. D, and F *vs*. D. Samples were prepared as described in the Sample preparation and Irradiation and dosimetry sections. Males of the required groups were split into two groups. The males of one of the halved groups were fed with 0.4% rhodamine B (Sigma Aldrich, 95% dye content) in 10% sucrose solution, as described by Johnson et al. [22] to mark sperm, whereas the other half was not marked.

For each competitive mating cross, 10 marked males from one treatment group and 10 unmarked males from a second treatment group were transferred to a 60 × 60 × 60 cm cage (Bugdorm^®^). Ten virgin females were subsequently added to the cage and were left to mate for 3 h, as recommended by Li et al. [27]. Females from each mating cross were then removed and kept frozen for later dissection. A second cross was then set up using males from the same two treatment groups, but with reciprocal marking status. A competitive mating cross of marked and unmarked males that were not irradiated served as controls to assess whether the marking itself had an effect on competitiveness. Females were chilled and dissected under a steromicroscope and the spermathecae removed and viewed under a fluorescence stereomicroscope (Olympus BX41, Tokyo, Japan) using an RFP1 filter to determine insemination status and the presence/absence of Rhodamine B. Four biological repetitions were performed for each cross.

### Statistical analysis

All statistical analyses were performed in R (version 4.1.0) using RStudio (RStudio, Inc. Boston, MA, USA, 2016). Generalized Linear Mixed Models (GLME, lme4 package) were used with the appropriate distribution family.

Male flight ability data were analyzed as response variable, treatment (6 levels: Treatment groups A–F) as fixed effect, and the repetition nested with technical repetition as a random effect considering each specific experiment.

Mixed Effects Cox Models (“coxme” function in “survival” package) fit by maximum likelihood with mosquito time to death as response variable, treatment (6 levels: Treatment groups A–F) as fixed effects, and repetition as a random effect, were used to analyze the survival of mosquitoes following the treatment in each specific experiment. Survival graphs were built using the packages “survival”, “ggplot2”, and “ggpubr”. Multiple comparisons using the “emmeans” function (in package ‘emmeans”) were performed to observe differences between specific treatment groups.

For the competitiveness tests, the effect or marking was first analyzed to ensure there was no effect. Data were then analyzed per mating cross separately (2 levels: treatment 1 and treatment 2), regardless of marking status using binomial models.

The full models were checked for overdispersion using Bolker’s function [4] (in package bblme). A *p*-value of less than 0.05 was used to indicate statistical significance in all cases.

## Results

### Dosimetry

The dosimetry confirmed that all doses received lay within a 3.07% error range (calibration MD film lot# 1222001; 2021.12.13).

#### Assessment of induced sterility

All irradiation treatments resulted in sterility levels beyond 99% in relation to non-irradiated controls (induced sterility). A dose of 70 Gy (group B) administered at once resulted in expected low levels of residual ferility of 0.007 ± 0.0026, whereas all fractionated doses (groups C–F with a total of 70 Gy) resulted in full sterility (100%), no matter the split dose proportions nor the number of days between exposures. There was a clear difference in induced sterilty after acute doses of 70 Gy and all fractionated exposures (χ^2^ = 11.060, *df* = 3, *p* < 0.0001).

#### Assessment of longevity

**Figure 1:**
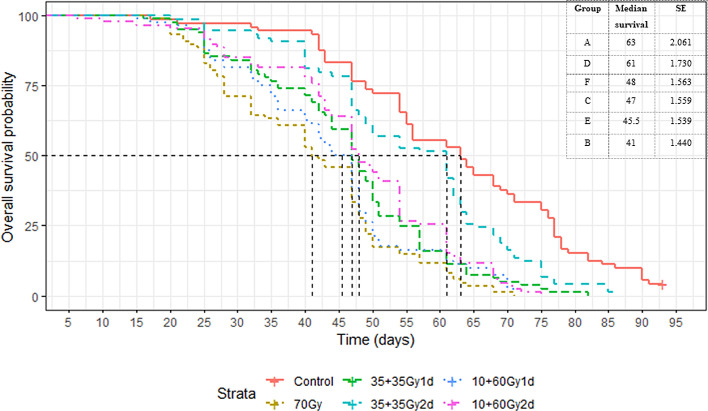
Survival curves of *Ae. aegypti* males sterilized with one acute dose or fractionated dose with 1- or 2-day intervals compared to untreated males. Table: Median survival (in days) of males in treatment groups A–F from highest to lowest.

Overall, non-irradiated control groups (A) lived longer than males in all other treatment groups (B–F) (*p* < 0.001), although group D was only slightly different from the Control (*p* = 0.012) (Fig. 1). Fractionation with a 1-day rest between exposures was not better than an acute 70 Gy dose, no matter how the dose was split (C *vs.* B: *p* = 0.079; E *vs*. B: *p* = 0.682), although the trend was still that the males from Group B (acute 70 Gy dose) performed the worst overall, especially after the first 3 weeks (Fig. 1). Fractionation with a 2-day rest between exposures was better than an acute dose, no matter how the dose was split (D *vs.* B: *p* = 0.001; F *vs.* B: *p* = 0.025). Two-day rest between exposures produced longer-lived males, no matter how the dose was split (D *vs.* C: *p* = 0.0025; D *vs.* E: *p* = 0.001). With a 2-day rest, the dose split into 35 + 35 Gy was more beneficial in terms of longevity than 10 + 60 Gy (D *vs.* F: *p* = 0.009; D *vs.* E: *p* < 0.001). The 1- or 2-day interval in the 10 + 60 Gy groups showed no difference in survival (F *vs.* E: *p* = 0.586). There was also no difference in the 1-day interval groups (C *vs.* E: *p* = 0.843). The full results of the multiple comparisons can be found in the Supplementary file.

#### Assessment of flight ability

**Figure 2:**
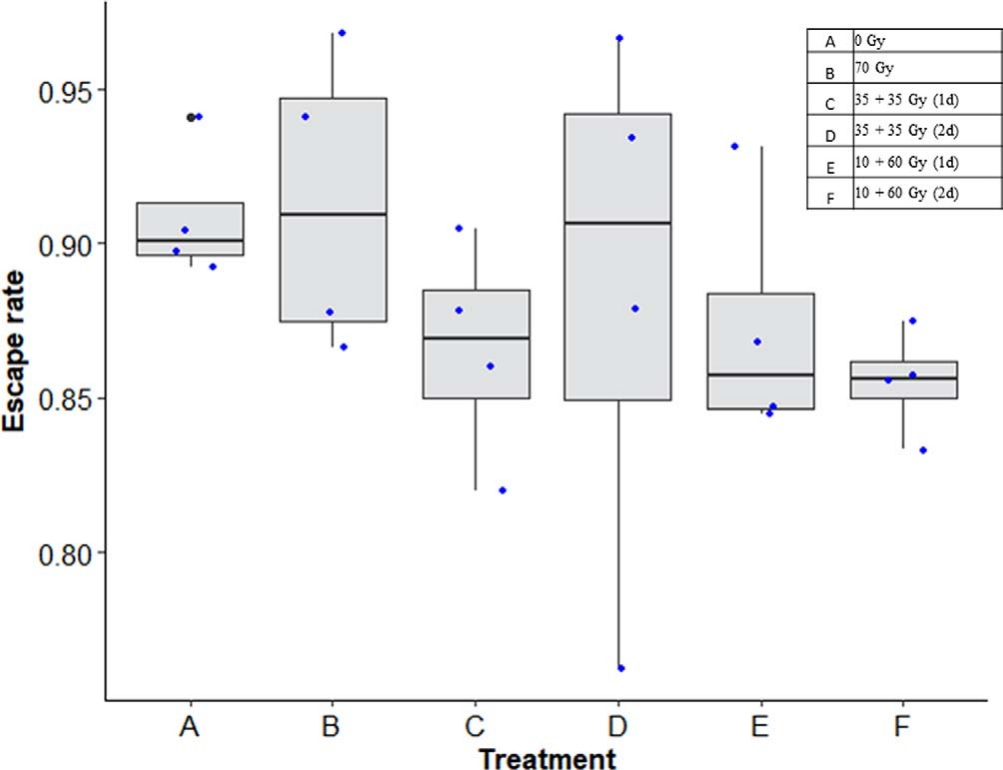
Escape rates of males irradiated with acute dose *vs*. fractionated dose with 1- or 2-day intervals, compared to non-irradiated control males.

Overall, the treatment had only a marginal effect on flight ability (χ^2^ = 10.309, *df* = 5, *p* = 0.0669). However, treatment “F” (10 + 60 Gy, 2-day interval) had a lower escape rate (Fig. 2, *p* = 0.0229).

#### Assessment of mating competitiveness

When pooling data from 70 Gy acute dose treatments and all fractionated dose treatments, the competitiveness was higher in fractionned treatments (*z* = −3.872; *p* = 0.0001). Only the 35 + 35 Gy fractionation treatment showed better competitiveness than the single 70 Gy dose (Table 2, Cross 1). Males irradiated with a 2-day interval between exposures were equally competitive regardless of the way the dose was split (Table 2, Cross 4). The marking status had no impact on competitiveness (Table 2, Cross 5).

**Table 2:** Competitivness index (C) of males sterilized by acute dose *vs*. fractionated dose, with 1- or 2-day intervals, and C of non-irradiated controls marked with Rhodamin B (Rhod+) or without marking (Rhod−).

Cross #	Treatment 1	Treatment 2	C (of Tx 1)	C (of Tx 2)	Estimate	*SE*	*z* value	Pr(>|*z*|)
1	70 Gy	35 + 35 (1)	0.304	**0.696**	−0.7732	0.349	−2.216	0.0267*
2	70 Gy	10 + 60 (1)	0.353	**0.647**	−0.5705	0.347	−1.644	0.1
3	35 + 35 (1)	35 + 35 (2)	0.369	**0.631**	−0.4964	0.339	−1.465	0.143
4	10 + 60 (2)	35 + 35 (2)	0.542	0.458	0.1625	0.3299	0.493	0.622
5	Control Rhod+	Control Rhod−	0.496	0.504	−0.05407	0.32892	−0.164	0.869

Values in bold represent a marked increase.

* indicates statistical significance.

## Discussion

This study was initiated with the aim of assessing the impact of radiation dose fractionation on *Aedes* male quality, as to date, no reports describing the effects of dose fractionation in mosquitoes in general are available. The fractionated dose of 70 Gy in two equal parts of 35 + 35 Gy was chosen following methods described in most historical studies on other insect species, and thus two equal medium doses seemed appropriate for this initial experiment. The second strategy of administering a low (10 Gy) dose, followed by a second higher (60 Gy) dose was based on the hypothesis that the initial low dose could serve as sort of “preconditioning”, whereby the cellular repair mechanism is stimulated, and may protect against excess somatic damage in the second exposure. A dose of 10–15 Gy alone has been shown to improve longevity in mosquitoes due to radiation hormesis compared to unirradiated males [1, 15, 19, 40]. To avoid prolonging the male production duration in an SIT facility, no more than 2 fractionated doses were considered for this study. Nor were recovery periods of more than 2 days considered between exposures, as it has been recommended to release the sterile males at around day 4 or 5 at the peak of their flight and mating activity, after which the flight ability begins to decline [29]. One and 2 days were selected as intervals also to ensure that there was sufficient time for the males to recover not only from the effects of the first irradiation, but also from the stress of handling before and during exposures, as it has been shown that, for example, flight ability is restored when males are given 1–2 days of rest post-exposure [29]. Selecting the length of intervals beween exposures is important and the ideal timing is not known for this species. The various publications describing dose fractionation studies in insects all have different intervals and number of exposures. A 4-hour interval between radiation doses allowed for some tissue recovery in the cotton leaf worm, whereas 2 h did not [37]. Increasing interval duration in tsetse flies from 1–2 days to 5 days also allowed recovery of chromosome damage and thus resulted in higher fertility rates in irradiated males [35]. Two doses with either 1 day, or 2 day intervals, or 3 doses were administered to West Indian sweet potato weevils (*Euscepes postfasciatus*) where it was found that fractionating the irradiation dose prolonged mating propensity significantly [25]. Other studies selected other intervals: 3 doses over 1–3 days for the Indian meal worm *Plodia interpunctella*, [5], 2, 3, or 4 equal doses with 2 h intervals in the spotted bollworm *Earias vitella*, [33], and 5 fractions with intervals of 1 min, 10 min, 1 h and 1 day in the grain beetle *Calandra granaria* [21]. Why these interval durations or number of fractions were selected was not clearly explained in most of the articles.

In our study, the acute sterilizing dose of 70 Gy achieved the expected sterility level of > 99%, with a few eggs hatching only, whereas the same dose fractionated resulted in 100% sterility with no eggs hatching in any of the batch samples, in all repetitions. This was unexpected as most other studies on dose fractionation in insects found that splitting doses resulted in less sterility than the equivalent acute dose [1, 5, 21]. However, Vreysen and Van der Vloedt [35] found that fertility increased when the interval durations increased, but was still less than that of males irradiated with an acute dose. Shantaram et al. [33] reported that sterility induced in the spotted bollworm (*Earias vittella*) was the same in males irradiated with an acute or fractionated dose, whereas other lepidopteran species presented reduced sterility levels following dose fractionation. A possible explanation is that male spotted bollworms emerge with a full set of sperm and there is no further multiplication of spermatogonia. One hypothesis is that sterility levels in some insects are significantly influenced by the timing of radiation exposures, depending on the process and timing of spermatogenesis occurring. If spermatids are fully formed, the effects of irradiation in either one acute dose, or several fractionated doses may not affect the final sterility level. In mature sperm of *Drosophila*, there was no effect of exposure to acute or chronic doses while in spermatids, increased genetic damage was observed when the dose was split [2], and thus increased sterility, as was observed in this study. The authors of the study proposed that oxygen was somehow released in the cellular components between the radiation doses, and thus increases radiation damage during the second dose. The observation that there was less biological damage with dose fractionation in argon than when oxygen is present supports this hypothesis. This notion is supported by Haynes et al [17] who suggested that fractionation or lowering dose rates may allow regeneration of sub-lethal cell damage, but increasing the number of fractions will reverse the beneficial effects; i.e., repeated radiation doses cause cells that were radioresistant due to hypoxia during previous doses to reoxygenate, and thus become 2–3 times more radiosensitive in subsequent exposures. Another possibility is that the chromosome breakage and/or repair mechanisms are affected, and this in turn depends on the stage of spermatogenesis. In sperm reaching maturity, a higher (subsequent) dose may be needed to reach the target sterility. In any case, it seems that spermatids and spermatozoa have different radiotolerance [35]. In a study in mice, Leonard and Deknudt [26] separated two fractionated doses by increasing time intervals. They concluded that the translocations caused by the second exposure were not all affected by or related to the damage caused by the first exposure, and that the fractionated interval effect was more related to the cell cycle; i.e., the second dose was either received by a radiosensitive or radioresistant stage of the cell cycle.

Although the historical publications reviewed in this study have reported differing effects of fractionation intervals on sterility levels and suggest different hypotheses on why this is the case, most studies agree that dose fractionation improved one or more male biological quality parameters. Few have reported no or negative effects. However, it is important to note that the number of fractions and time intervals are important for the outcome and thus changing these variables may have resulted in a better outcome in the particular insect studied. In our study, splitting the sterilizing dose for *Ae. aegypti* males into two fractions, with an interval of 1 or 2 days, improved longevity in all treatment groups as compared to the males irradiated with one acute dose. The trend showed that males receiving 2 days rest between doses survived longer than those with only 1-day rest. In both the 2-day interval groups and the 1-day interval groups, the males exposed to 2 equal doses of 35 Gy survived longer than those irradiated with a low dose (10 Gy) followed by a high dose (60 Gy). This may be because 60 Gy is still a relatively high dose, and not much reduced from the total acute dose of 70 Gy.

There was no difference observed in flight ability between males subjected to acute or fractioned doses. All treatment groups performed equally as compared to non-irradiated control groups, except treatment group F. This result suggests that subjection to one high dose, *or* the double handling, *or* only having one recovery day is tolerable in terms of flight ability; however, when all three factors are combined, this reduces the overall male quality, which is reflected by the reduced escape rates [29]. Although not statistically significant, the trend was that the double handled males all had the lowest recorded escape rates (C–F), when compared to the low scores of the males handled only once (A and B), suggesting that stress from handling can be more detrimental than irradiation itself [9].

Overall, there was no observed difference between males receiving two equal medium doses, or one low then one high, except for males exposed to two doses of 35 Gy, which showed better competitiveness. A 2-day interval provided better recovery than a 1-day interval both in the longevity and flight ability tests.

## Conclusions

Different insect species may be more susceptible to acute doses of irradiation, and these may benefit from fractionation. Others may be more sensitive to increased handling and stress. Handling of adult mosquitoes in preparation for irradiation includes briefly chilling the adults and aliquoting batches into separate tubes, (or compacting large numbers of chilled adults for mass irradiation), transportation to and from the irradiation facility and then back to the insectary. Considering that males subjected to fractionated doses had double handling and still performed better in the survival assays and maintained this trend in competitiveness tests showed that dose fractionation does seem to reduce overall radiation damage in this species. However, the question still remains whether the biological benefits of dose fractionation outweigh the additional labor and thus reduced production efficiency in mosquito SIT programmes. It would be essential to assess the competitiveness of the sterile males resulting from the various fractionation treatments in the field, and the duration of any improved competitiveness over several days as was done, for instance, for the West Indian sweet potato weevil [25]. Other combinations of split doses and recovery periods may result in a better outcome and may warrant the extra efforts. The marginal improvements in longevity and mating competitiveness in the laboratory suggest that dose fractionation into two equal doses may only be recommended for this mosquito species if these quality improvements are confirmed in the field.

## Figures and Tables

**Figure S1.**
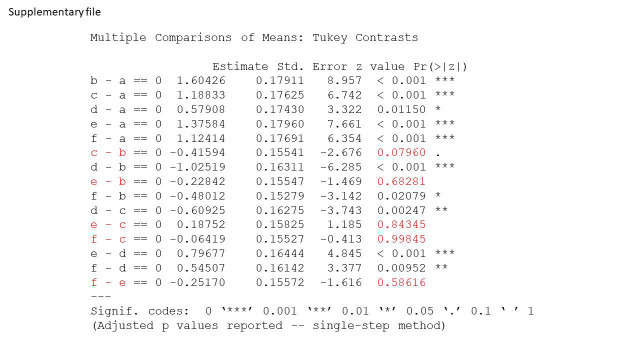
Multiple comparisons of means: Tukey contrasts.

## Data Availability

The datasets used and/or analyzed during the current study, including all dosimetry reports, are available from the corresponding author upon reasonable request.

## Supplementary Materials

The Supplementary materials of this article are available at https://www.parasite-journal.org/10.1051/parasite/2023005/olm
